# Research on the Effect of Vibrational Micro-Displacement of an Astronomical Camera on Detector Imaging

**DOI:** 10.3390/s24031025

**Published:** 2024-02-05

**Authors:** Bin Liu, Shouxin Guan, Feicheng Wang, Xiaoming Zhang, Tao Yu, Ruyi Wei

**Affiliations:** 1 Xi’an Institute of Optics and Fine Mechanics, Chinese Academy of Sciences, Xi’an 710119, China; liubin@opt.cn (B.L.); 3140101019@zju.edu.cn (S.G.); wangfeicheng@opt.ac.cn (F.W.); yutao@opt.ac.cn (T.Y.); 2 National Astronomical Observatories, Chinses Academy of Sciences, Beijing 100012, China; xiaomingzhang@bao.ac.cn; 3 School of Electronic Information, Wuhan University, Wuhan 430072, China; 4 Hubei Luojia Laboratory, Wuhan 430079, China; 5 Wuhan Institute of Quantum Technology, Wuhan 430023, China

**Keywords:** vibration, resonance, displacement, cooling, cameras

## Abstract

Scientific-grade cameras are frequently employed in industries such as spectral imaging technology, aircraft, medical detection, and astronomy, and are characterized by high precision, high quality, fast speed, and high sensitivity. Especially in the field of astronomy, obtaining information about faint light often requires long exposure with high-resolution cameras, which means that any external factors can cause the camera to become unstable and result in increased errors in the detection results. This paper aims to investigate the effect of displacement introduced by various vibration factors on the imaging of an astronomical camera during long exposure. The sources of vibration are divided into external vibration and internal vibration. External vibration mainly includes environmental vibration and resonance effects, while internal vibration mainly refers to the vibration caused by the force generated by the refrigeration module inside the camera during the working process of the camera. The cooling module is divided into water-cooled and air-cooled modes. Through the displacement and vibration experiments conducted on the camera, it is proven that the air-cooled mode will cause the camera to produce greater displacement changes relative to the water-cooled mode, leading to blurring of the imaging results and lowering the accuracy of astronomical detection. This paper compares the effects of displacement produced by two methods, fan cooling and water-circulation cooling, and proposes improvements to minimize the displacement variations in the camera and improve the imaging quality. This study provides a reference basis for the design of astronomical detection instruments and for determining the vibration source of cameras, which helps to promote the further development of astronomical detection.

## 1. Introduction

In the field of astronomical detection, ground-based observation is an indispensable means of observation [1]. Since the observation target is very far away from Earth, the detector can receive a very limited signal light, so during the detection process, the camera should be kept as stable as possible so as to avoid an image shift and to achieve better imaging quality [2]. Vibration is one of the destabilizing factors for cameras in the detection process [3]. Cameras undergo external vibration and internal vibration; external vibration mainly includes environmental vibration that transmits through the mechanical structure of camera, and internal vibration refers to the camera’s internal moving parts generating a force that leads to camera vibration [4]. External vibration and internal vibration occur together in the whole camera, which cause the camera to produce a resonance effect, and thus, produce a greater vibration impact.

In the field of astronomy, since the target light all comes from stars outside of Earth, and in some cases, far outside the Milky Way, it usually takes a long integration time to capture the weak light signals, and vibrations cause micro-displacements in the camera itself [5]. If there is relative motion between the camera and the subject during the exposure process, image shift mismatch or imaging blurring can occur, reducing detection accuracy [6]. Camera displacement variation is one of the most important factors influencing the accuracy and stability of astronomical surveys [7]. This paper takes the Andor_iKON_L_936 camera [8] as the research object, analyses the micro-displacement produced by the camera due to the vibration that exists in its working process, and compares and verifies the experimental results, and finds that the change in the camera displacement in the water-cooled mode is significantly smaller than that in the air-cooled mode. The effect of camera displacement on optical imaging is analyzed, and a corresponding correction scheme is proposed to improve the stability of the camera. The conclusions of this paper provide a reference basis for the design of astronomical detection instruments.

## 2. Camera Shift Analysis

### 2.1. External Influences

During the operation of a camera, external vibrations mainly come from ambient vibrations and resonance effects. Ambient vibrations act directly on the camera itself, and even on a vibration-isolated optical platform in a laboratory, micro vibrations are still present. In this paper, all experiments were carried out in a standard optical laboratory using an AEROTECH nanopositioning stage system to measure displacement changes due to external vibrations acting on the camera, which has nanometer-level accuracy and can accurately measure displacement changes in a given direction.

**Figure 1:**
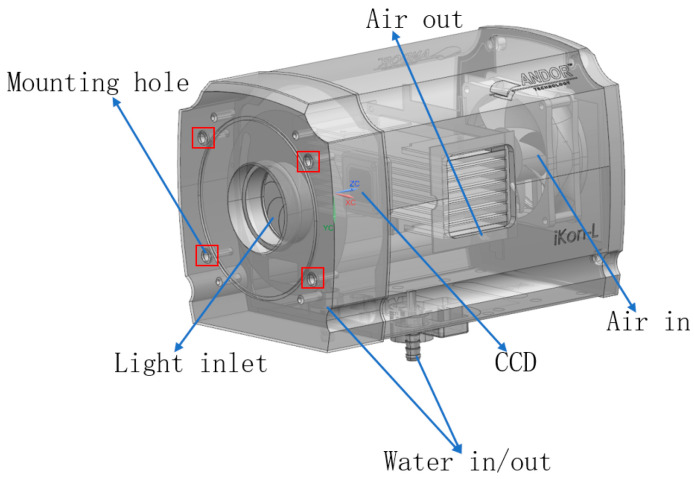
Structural model of the camera.

**Figure 2:**
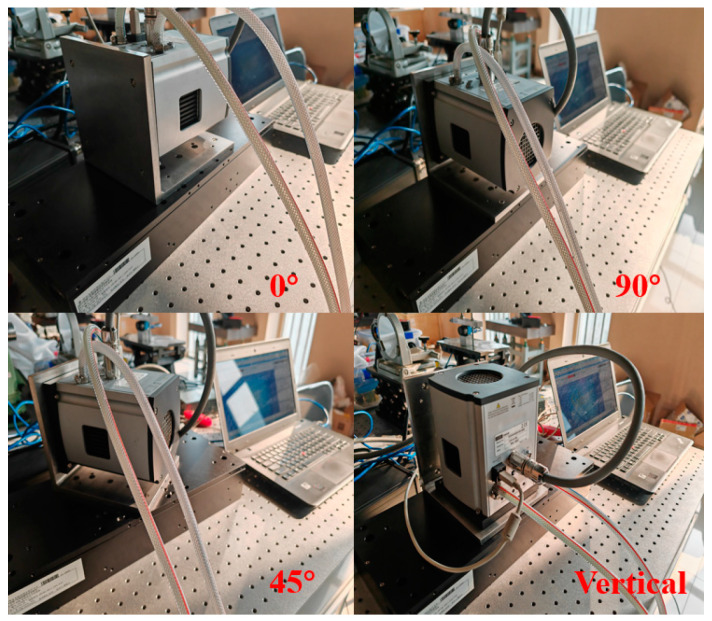
Camera shift experiment.

As shown in Figure 1, the camera used in the experiments of this paper is an Andor_iKON_L_936 CCD camera, which has fixed threaded holes reserved on the front surface of the light-entry port. To simulate actual use, an L-shaped adapter plate was designed to secure the camera to the AEROTECH nanopositioning platform. At rest, the readings from the nano-displacement stage at this point can be thought of as the amount of displacement of the external ambient vibration translated into the camera itself. By changing the mounting attitude of the camera, the translation of external environmental vibrations into the displacement of the camera itself in different directions is simulated. Figure 2 shows the camera fixed on the nano-displacement platform in different attitudes.

The camera is fixed on the nano-displacement platform through the L-shaped adapter plate. Since the nano-displacement platform can only test the displacement change in a specified direction at a single time, the displacement of the environmental vibration acting on the camera in different directions is obtained by changing the relative angle between the direction of the camera and the guide rail of the nano-displacement platform. In this experiment, each 45° rotation is taken as a sampling point, each test point is repeated three times, and the average value is taken as the displacement change value in the state where the center line of the optical inlet is parallel to the mounting surface of the nano-displacement platform. The centerline of the camera’s optical inlet and the nano-displacement stage mounting surface are in a vertical state, alone, as a testing attitude. The test results of displacement changes due to environmental factors are shown in Table 1.

### 2.2. Internal Influences

Cameras operate with deep cooling of the CCD to reduce dark-current noise [9]. The cooling of the CCD generally occurs through TE cooling [10], such as in the Andor_iKON_L_936 camera used in this paper, which employs a five-stage TE cooler. The TE cooler functions through the thermoelectric effect of the semiconductor, which, at the front end of the cooler, produces a low temperature, and at the back end, a high temperature. The back end of the cooler enables timely heat dissipation. If the front and rear ends of the surface of the cooler have too high a temperature difference, it will affect the cooling efficiency of the TE cooler. The Andor camera’s five-stage TE cooler allows the CCD to operate at temperatures as low as −100 °C. The heat generated at the back end of the TE cooler is dissipated by the camera’s air- or water-cooled modules using heat exchange.

The camera’s air-cooling mode is based on the high-speed rotation of the cooling fan, which drives gas from the vicinity of the fan into the camera’s interior, and then, out of the camera through the air vents on both sides of the camera, etc., for the circulation of the gas. The low-temperature gases in the cycle are convectively exchanged inside the camera and at the back end of the TE cooler to lower the temperature of the TE cooler. The fan blades are subjected to large aerodynamic forces generated by the action of airflow in a high-speed rotating operating environment [11], and the aerodynamic loads on the blades can be expressed as follows:

(1) $$P_{a}=\frac{2\pi r}{z}\left(P_{1}-P_{2}+\rho_{2}C_{2a}^{2}-\rho_{1}C_{1a}^{2}\right)$$

(2) $$P_{n}=\frac{2\pi r}{z}\left(\rho_{2}C_{2a}C_{2n}-\rho_{1}C_{1a}C_{1n}\right)$$

$P_{a}$ is the axial aerodynamic force at a point in the direction of the leaf height; $P_{n}$ is the aerodynamic circumferential force at a point along the leaf height direction; z is the number of fan blades on the fan disc; r is the distance of the point from the rotation axis; $P_{1}$ and $P_{2}$ are the pressures at the inlet and outlet of the blade; $\rho_{1}$ and $\rho_{2}$ are the airflow densities at the inlet and outlet of the blade; $C_{1a}$ and $C_{2a}$ are the axial and circumferential airflow rates at the blade inlet; and $C_{1n}$ and $C_{2n}$ are the axial and circumferential airflow rates at the blade outlet.

**Figure 3:**
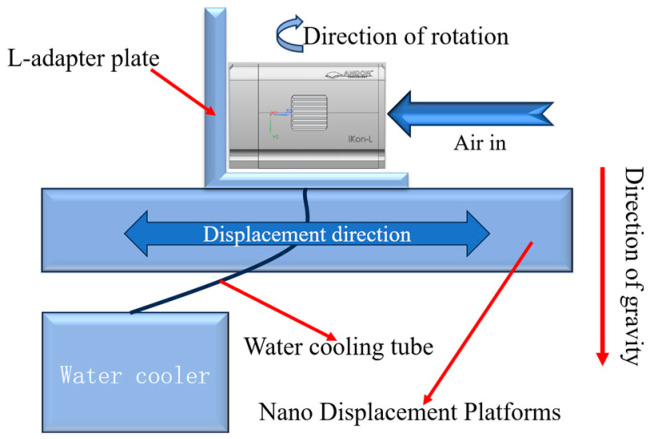
Experimental principle of nano-displacement platform.

As shown in the experimental principle of Figure 3, the camera is fixed to the nano-displacement platform through the L-type adapter plate, and in the working state of the camera, the camera air-cooling mode is turned on, and the displacement change in the nano-displacement platform is observed and recorded; then, it is considered that the numerical change in the nano-displacement platform is the displacement change in the camera caused by the air-cooling mode. By changing the relative angle between the camera and the direction of the nano-displacement platform guide, the air-cooled mode causes the displacement of the camera in different directions. In this experiment, under the state that the center line of the optical inlet is parallel to the mounting surface of the nano-displacement platform, each rotation of 45° is taken as a test point; the test is repeated three times for each test point, and the average value is taken as the change value of the displacement. The centerline of the camera’s optical inlet and the mounting surface of the nano-displacement stage in a perpendicular state are treated separately as a test state. The test results of the displacement changes due to the air-cooling mode are shown in Table 2.

Water-circulation refrigeration uses a chiller to introduce cooling water of a certain temperature into the cold head [12], and the cooling water circulates fully in the cold head, where the end face of the cold head is in contact with the rear face of the TE cooler, displacing the heat using heat conduction, and then, taking the heat out of the camera through the circulating water. The process of circulating cooling water through the camera’s pipework and cold head generates axial and circumferential loads on the mechanical structure.

(3) $$\sigma_{z}=\frac{E}{\left(1+\nu \right)\left(1-2\nu \right)}\left[\left(1-\nu \right)\frac{\partial u_{z}}{\partial_{z}}+\nu \frac{1}{r}\frac{\partial }{\partial r}\left(ru_{z}\right)\right]$$

(4) $$\sigma_{\phi }=\frac{E}{\left(1+\nu \right)\left(1-2\nu \right)}\left[\left(1-\nu \right)\frac{\partial u_{\phi }}{\partial_{R}}+\nu \frac{1}{r}\frac{\partial }{\partial r}\left(ru_{\phi }\right)\right]$$

$\sigma_{z}$ is the axial stress of the pipe; $\sigma_{\phi }$ is the circumferential stress of the pipe; R is the fluid diameter; r is the pipe diameter; ν is the fluid velocity; $u_{z}$ is the axial component of the fluid velocity; $u_{\phi }$ is the circumferential component of the fluid velocity; and E is the Young’s modulus of the pipe material.

Also, as shown in the experimental principle of Figure 3, the camera is fixed to the nano-displacement platform through the L-type adapter plate, and in the working state of the camera, the water-cooling mode of the camera is turned on, and the displacement change in the nano-displacement platform is observed and recorded; then, it is considered that the numerical change in the nano-displacement platform is the displacement change in the camera caused by the water-cooling mode. By changing the relative angle between the camera and the direction of the nano-displacement platform guide, the water-cooling mode causes displacement of the camera in different directions. In this experiment, under the state that the center line of the optical inlet is parallel to the mounting surface of the nano-displacement platform, each rotation of 45° is taken as a test point, and the test is repeated three times for each test point, and the average value is taken as the change value of the displacement. The centerline of the camera’s optical inlet and the mounting surface of the nano-displacement stage in a perpendicular state are treated separately as a test state. The test results of displacement changes due to the water-cooling mode are shown in Table 3.

**Figure 4:**
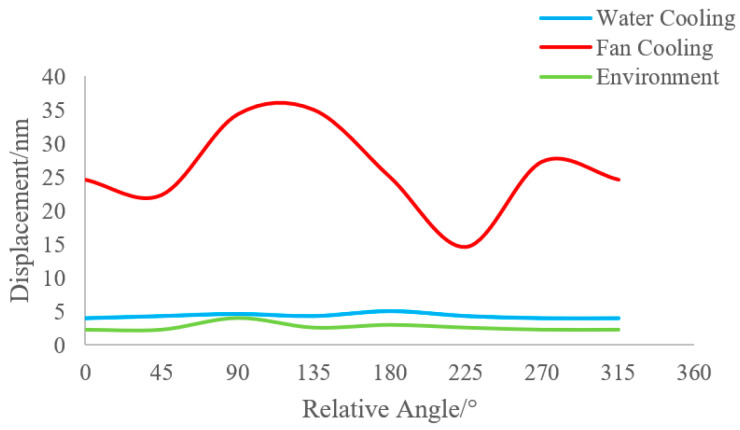
Comparison of camera shift in different states.

From the experimental data in this section, it can be seen that ambient vibration, water-cooled mode, and air-cooled mode all cause displacements in different directions on the camera. The effect of ambient vibration on the camera is invariant and is coupled with both the water-cooled and air-cooled modes of operation. The water-cooled mode reaches a steady-state flow in the water-circulation line after some time, so it causes minimal displacement changes to the camera. In the air-cooled mode, the cooling fan is rotating; on the one hand, the airflow movement will generate force on the camera, and on the other hand, the vibration generated by the fan rotation will be mechanically transferred to the camera, and it is more likely to generate a resonance effect on the camera. Figure 4 compares the changes in the displacement of the camera due to environmental factors, the water-cooled mode, and the air-cooled mode.

### 2.3. Vibration Analysis

**Figure 5:**
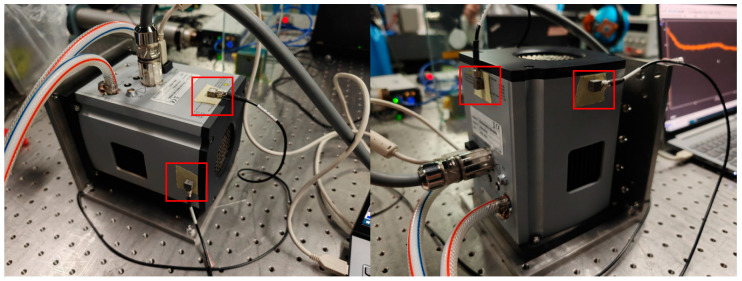
Camera vibration experiment.

To further analyze the causes of camera displacement, this paper designs a vibration frequency measurement experiment. As shown in Figure 5, the Andor_iKON_L_936 CCD camera was fixed on an optical platform through an L-shaped adapter plate in the same state, accelerometers [13] were stuck to different points of the camera, and the time-domain acceleration data of the camera were recorded in the camera’s resting state, water-cooled mode, and air-cooled mode, respectively. The data were analyzed spectrally to obtain information about the camera vibrations in the frequency domain.

**Figure 6:**
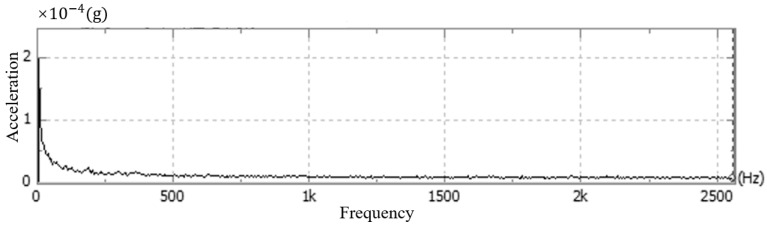
Camera vibration at rest.

**Figure 7:**
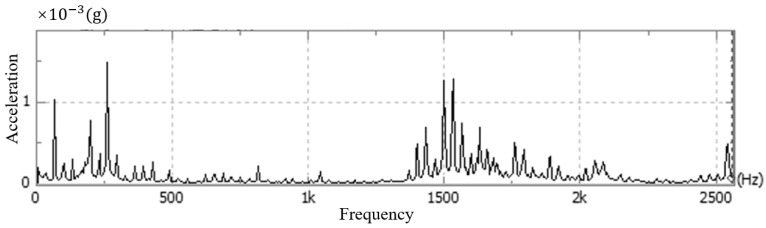
Camera vibration in air-cooled mode.

**Figure 8:**
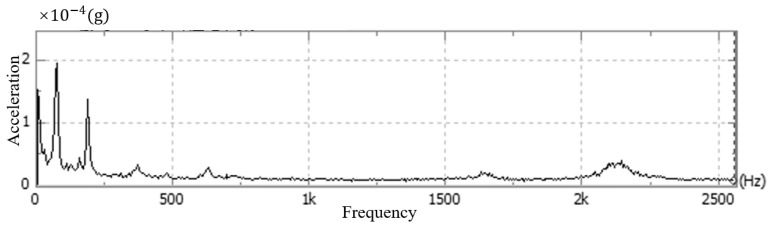
Camera vibration in water-cooling mode.

**Figure 9:**
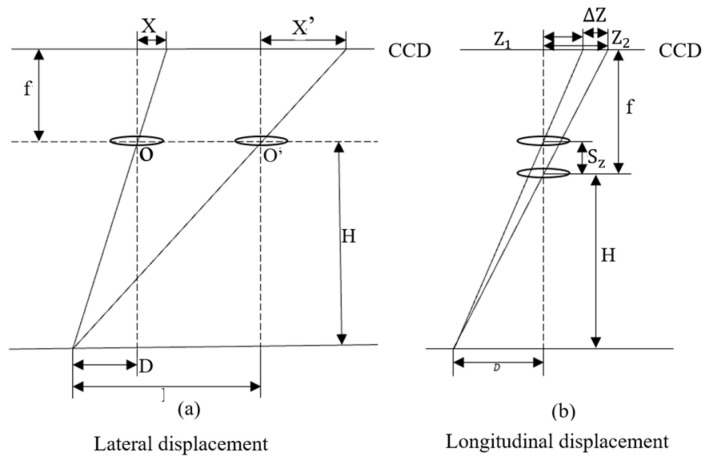
Effect of changes in displacement in different directions.

Andor cameras are fixed singly, usually with screws placed through the four round holes on the surface of the camera’s light-entry port. As shown in Figure 5, the camera is generally fixed using the flange at the optical inlet and the structural member connected by screws, and the mounting direction of the optical inlet centerline is parallel to the optical platform. Therefore, in this paper, the above vibration test experiments are carried out for the common fixation methods, respectively, for the stationary state, air-cooled state, and water-cooled state of the camera. This experiment uses the CONIV’s acceleration measurement system, which includes acceleration sensors, data acquisition cards, and other devices. The comparison of the experimental results is shown in Figure 6, Figure 7, Figure 8 and Figure 9 below.

As shown in the above figure, it can be seen that under the same constraints, the acceleration amplitude of the camera is stable at rest, and in the frequency domain range, the acceleration amplitude is lower and varies steadily. When the camera is turned on in the water-cooled mode, the acceleration amplitude of the camera measurement point in the frequency range changes significantly less compared with the air-cooled mode, and the maximum amplitude of the acceleration is about one-tenth of that of the air-cooled mode, which is in line with the results of the displacement experiments in this article.

The above experimental results show that since the cooling water flowing in the water-cooled piping reaches a steady-state flow after circulating for a certain period, the pressure generated on the water-circulation lines in the camera is small and very stable. In the air-cooled mode, the turbulent flow of gas will generate pressure on the camera with a certain periodicity, and when the frequency of the pressure is close to the intrinsic frequency of the camera itself, a large resonance phenomenon may occur, which affects the imaging. In addition, the mechanical vibration of the fan as it rotates is transmitted directly to the camera itself, which can affect camera displacement changes.

## 3. Imaging Quality Analysis

The position of the picture element changes as a result of the CCD camera’s displacement during long exposure, which has a negative impact on imaging quality [14]. The graphic below illustrates the impact of shifting the camera’s d The authors believe that the position of Figure 9 should not be changed because it is an explanation of the image shift and corresponds mainly to section III of the text.isplacement in various directions on imaging. Transverse and longitudinal displacements are presented as examples.

The image point change due to the change in transverse displacement can be expressed as follows:

(5) $$\Delta X=X^{\prime }-X=(D^{\prime }-D)\cdot \frac{f}{H}=\Delta D\cdot \frac{f}{H}$$

The change in image point position due to a change in longitudinal displacement can be expressed as follows:

(6) $$\Delta Z=Z_{2}-Z_{1}=\frac{\mathrm{Df}\cdot S_{z}}{H\left(H-S_{z}\right)}$$

As shown in Figure 9, during the change in lateral displacement, X is the initial position of the image point; $X^{\prime }$ is the position of the image point after being affected by vibration; D is the initial position of the object point; $D^{\prime }$ is the relative position of the object point after being affected by vibration; f is the focal length of the camera; and H is the shooting height. In the longitudinal displacement change, the initial image point distance from the center of the image plane is $Z_{1}$, the exposure time affected by the vibration of the camera moving distance is $S_{z}$, and the image point distance from the center of the image plane is $Z_{2}$.

The main sources of camera displacement changes brought about by the refrigeration unit in operation are fluid–solid coupling vibration brought about by fluid pressure, environmental vibration, mechanical vibration of the refrigeration fan, and its harmonic vibration. For the passive control of vibration, the principle is to avoid resonance points and reduce the intensity of vibration by reducing the amplitude of the corresponding vibration force, changing the ratio between the frequency of the force applied to the camera and the intrinsic frequency of the camera, or increasing the structural damping of vibration-prone locations. For water-cooled pipelines, energy storage structures can be set up in the pipeline, which can absorb the impact force generated by the water; in addition, they can also be used to increase the overall rigidity of the water-cooled pipeline system through a reasonable arrangement of pipeline supports, to improve their resistance to deformation. In the case of air-cooled systems, the mechanical vibration caused by fan cooling can be reduced by increasing the strength of the material at the end of the fan blades, and by installing flexible materials to isolate vibration at the location where the fan is connected to the camera. In addition, the frequency is changed by optimizing the speed of the fan, thus achieving a change in the ratio to the camera’s intrinsic frequency. At the same time, this can also enable some active vibration control; the principle is to detect the camera vibration and environmental vibration information through technical means to obtain the camera’s vibration law, and then, use the appropriate actuator output (reverse vibration wave or vibration force) to achieve direct camera vibration control. In the instrument design stage, the displacement changes caused by camera vibration may be along multiple directions, and technical means can be used to accurately measure the instrument vibration; additionally, algorithms can be used to correct the position of the image point at a later stage, which can also improve the imaging quality to a certain extent.

The displacement experiments show that the camera produces displacement changes of about 20–30 nm in the air-cooled mode and about 5 nm in the water-cooled mode. The amount of displacement produced in both modes appears to be very small, but in the astronomical realm, great distances magnify the effect that such displacement changes have on imaging. And for CCD cameras like the Andor_iKON_L_936, it has a pixel distribution of 100 percent, which means that there is no spacing between pixels on the CCD, which, in the field of astronomical spectroscopy, affects the spectral resolution of the instrument. In conclusion, the control of small displacement changes is of interest in hyperspectral, high-resolution applications.

For high-precision ground-based astronomical spectrometers, imaging blur is divided into image blur and image shift blur. The sources of image blur include atmospheric turbulence, structural errors in astronomical telescopes, etc., and most of the image blur can be corrected by algorithmic techniques. Shift blur is a change in the relative position between the light source and the camera due to vibration factors, and the stability of the instrument needs to be improved to limit this kind of error.

Camera micro-displacement produces a shifting blur during imaging, which can lead to an increase in the width of the spectral lines, making the measurement of spectral line spacing inaccurate and causing difficulties in spectral signature analysis. Micro-displacement causes changes in the intensity of the spectral signal, which reduces the signal-to-noise ratio, makes it difficult to detect weak signals, and may affect the accurate measurement of spectral features. Micro-displacement can lead to changes in the optical range length, and the optical range difference between the two interfering arms of a ground-based astronomical spectrometer based on the principle of interference may change, which ultimately affects the accurate analysis of spectral features. Micro-displacement may cause deformation or positional changes in optical elements, which may affect the focusing and transmission of the beam, which may lead to a loss of the collimation and accuracy of the spectrometer. Micro-displacement may cause errors between the optics and the calibration of the instrument, leading to calibration failures.

## 4. Results

In this paper, the camera displacements due to different cooling modes of the Andor_iKON_L_936 CCD camera were tested, and it was found that water-circulation cooling produces a much smaller change in displacement relative to fan cooling, almost one-tenth of that of the air-cooled mode. In addition, we conducted vibration tests in a laboratory environment, and the results show that the vibration generated by the air-cooled mode causes the camera to produce accelerations of higher amplitude and that the camera produces larger accelerations over a wider frequency range. The maximum magnitude of camera acceleration in the air-cooled mode is about ten times that in the water-cooled mode, and the conclusions are the same as in the displacement experiments.

We also analyzed the causes of imaging quality degradation caused by displacement and proposed solutions. The conclusions drawn in this paper provide an important reference basis for choosing a camera cooling method during the instrument design process, which helps to achieve high-quality astronomical or other remote sensing imaging.

In the field of astronomical detection, especially in the direction of astronomical spectra, it is necessary to use CCD cameras with higher resolution; the higher the resolution, the smaller the corresponding angular resolution of each image element, and the higher the requirements for the stability of the camera. In the astronomical field, CCD cameras have long exposure times, and small displacements during the exposure process can cause the degradation of imaging quality. It may affect the accuracy of the inversion of information such as the position, volume, and weight of the observed object. Also on the optical side, changes in camera displacement may cause the relative position of the optics on the focal plane to change, resulting in a change in optical range and introducing calculation errors.

For high-precision ground-based astronomical spectrometers, we have to consider the impact of vibration on their detection accuracy. In considering the impact of vibration, firstly, we have to consider the extent to which vibration in different frequency ranges affects the measurement results, so that corresponding measures can be taken all the time or compensated for. Secondly, it is necessary to consider the effect of the magnitude of the vibration on the spectrometer to determine whether it exceeds the tolerance range of the optical design in order to determine whether measures need to be taken to reduce it to an acceptable level. Finally, the transmission path of the vibration should be considered to determine where the most appropriate vibration isolation measures should be taken.

## Figures and Tables

**Table 1:** Camera displacement due to environmental vibration.

Relative Angle/°	0	45	90	135	180	225	270	315	Vertical
First recording/nm	2	2	4	3	4	3	2	3	2
Second recording/nm	3	2	5	2	2	2	3	2	2
Third recording/nm	2	3	3	3	3	3	2	2	2
Average/nm	2.3	2.3	4	2.6	3	2.6	2.3	2.3	2

**Table 2:** Camera shift due to air-cooled mode.

Relative Angle/°	0	45	90	135	180	225	270	315	Vertical
First recording/nm	23	21	36	34	25	13	27	24	12
Second recording/nm	26	24	34	36	26	16	29	26	12
Third recording/nm	25	22	33	35	24	15	26	24	13
Average/nm	24.6	22.3	34.3	35	25	14.6	27.3	24.6	12.3

**Table 3:** Camera shift due to water-cooling mode.

Relative Angle/°	0	45	90	135	180	225	270	315	Vertical
First recording/nm	3	3	4	3	4	4	4	3	3
Second recording/nm	4	5	5	5	6	5	3	5	3
Third recording/nm	5	5	5	5	5	4	5	4	2
Average/nm	4	4.3	4.6	4.3	5	4.3	4	4	2.6

## Data Availability

Data are contained within the article.
